# Broad Learning System under Label Noise: A Novel Reweighting Framework with Logarithm Kernel and Mixture Autoencoder

**DOI:** 10.3390/s24134268

**Published:** 2024-06-30

**Authors:** Jiuru Shen, Huimin Zhao, Wu Deng

**Affiliations:** College of Electronic Information and Automation, Civil Aviation University of China, Tianjin 300300, China; 2023022112@cauc.edu.cn (J.S.); wdeng@cauc.edu.cn (W.D.)

**Keywords:** Broad Learning System, Logarithm Kernel, adaptive weight calculation, Mixture Autoencoder, label noise learning, noisy data classification

## Abstract

The Broad Learning System (BLS) has demonstrated strong performance across a variety of problems. However, BLS based on the Minimum Mean Square Error (MMSE) criterion is highly sensitive to label noise. To enhance the robustness of BLS in environments with label noise, a function called Logarithm Kernel (LK) is designed to reweight the samples for outputting weights during the training of BLS in order to construct a Logarithm Kernel-based BLS (L-BLS) in this paper. Additionally, for image databases with numerous features, a Mixture Autoencoder (MAE) is designed to construct more representative feature nodes of BLS in complex label noise environments. For the MAE, two corresponding versions of BLS, MAEBLS, and L-MAEBLS were also developed. The extensive experiments validate the robustness and effectiveness of the proposed L-BLS, and MAE can provide more representative feature nodes for the corresponding version of BLS.

## 1. Introduction

In recent years, deep learning, as a research hotspot in the field of artificial intelligence, has achieved significant breakthroughs and widespread applications in various domains [1,2]. Although deep learning boasts powerful learning capabilities, its training process is extremely time-consuming due to its complex model structure and the iterative adjustment of numerous hyperparameters [3,4].

Against this backdrop, Chen et al. [5] proposed a neural network learning framework called the Broad Learning System (BLS) in 2018. It originated from the Random Vector Functional Link Neural Network [6]. BLS extends the width of neural networks to fit the data and has advantages such as a simple network structure and fewer model parameters [7,8,9]. BLS does not require iterative adjustment and has an extremely fast learning speed. Since its proposal, BLS has attracted widespread attention and has rapidly developed in both theoretical and applied research fields.

Researchers have further explored the excellent performance of BLS, leading to further developments in many challenging areas [10,11,12,13]. Chen et al. [14] developed a cascade structure, a recurrent structure, and a wide and deep combination structure based on BLS. Ye et al. [15] implemented image denoising by using cascaded BLS that connects feature mapping node groups and enhancement node groups in sequence. For chaotic time series prediction.

Furthermore, BLS demonstrates excellent performance in semi-supervised learning. For instance, Zhao et al. [16] developed a semi-supervised BLS (SS-BLS) that utilizes manifold regularization to obtain pseudo-labels for unknown data, thereby expanding the training set. Huang et al. [17] designed a generalized model with manifold regularization sparse features (BLS-MS), which utilizes latent information hidden in unlabeled samples for representation learning. Bhosle et al. [18] designed a deep learning CNN model for the recognition of the Devanagari digit. Deng et al. [19] developed a cluster clustering-based modular with a deep neural network for predicting flight arrival time.

The aforementioned BLS methods and their variants have shown good generalization performance and practical effectiveness, but these results are typically obtained under conditions where training samples are free from label noise. In recent decades, the rapid advancement of sensor technology has greatly raised the bar for sensor complexity, accuracy, and efficiency. The operating status of a sensor is critical to its health and reliability. However, during the data annotation process, factors such as sensor aging, human annotation errors, and environmental issues can degrade data quality, leading to incorrect labels, posing significant challenges to sensor operation. Addressing label noise is critical to improving the accuracy and reliability of sensor prediction and health management systems. The broad learning system employs ridge regression for output weights; although BLS uses one-hot encoding for sample labels, it is still highly sensitive to labeling noisy data. This has been elaborated on in detail [20]. Therefore, in the label noise environment, we urgently need a more robust BLS method.

In response to the above problems, in previous studies, Lu et al. [21] proposed a robust weighted least squares support vector machine. Jin et al. [22] combined the L1 and L2 norms and effectively optimized the BLS model using the augmented Lagrange multiplier method to enhance model robustness. Chu et al. [23] introduced several weighted penalty factors to enhance model performance, resulting in the design of a weighted BLS (WBLS). Liu et al. [24] utilized modal regression instead of least squares measurement to train a generalized network, which generated a diagonal weight matrix through optimization strategies for stronger noise penalties. A notable work in improving the robustness of BLS is the robust manifold BLS (RM-BLS) [25]. By introducing manifold embedding and random perturbation approximation, it aims to achieve robust mapping characteristics in certain specialized applications, such as predicting chaotic time series with noise. Zheng et al. [26] employed the maximum correlation entropy criterion to train network connection coefficients, achieving outstanding regression and classification performance in noisy environments. The graph regularized BLS introduced in [27] incorporates a target function based on maximum likelihood estimation, assigning appropriate weights to each sample for classifying data with label noise. In addition, other methods have also been proposed in recent years [28,29,30,31,32,33,34,35,36].

The above methods have achieved certain results for BLS, but there is still room for improvement. In this paper, the main contributions are listed as follows:

(1) Based on the Gaussian kernel, a novel function called Logarithm Kernel (LK) is constructed to effectively enhance the robustness of BLS under label noise conditions.
(2) A new robust broad learning reweighting framework (L-BLS) is designed by adding Logarithm Kernel (LK) to BLS for training output weights in order to significantly mitigate or eliminate the impact of label noise on BLS.
(3) A Mixture Autoencoder (MAE) is constructed to create more representative feature nodes in BLS for image databases with numerous features.
(4) MAEBLS and L-MAEBLS were developed to improve the expressiveness of the enhanced feature nodes of the corresponding BLS version and reduce their sensitivity to label noise.

The rest of this paper is organized as follows: Section 2 provides a brief review of BLS. In Section 3, we elaborate on the proposed methods. Section 4 presents the robustness analysis of the Logarithm Kernel (LK). Section 5 offers extensive experimental results and discussions. Finally, Section 6 concludes the paper.

## 2. Review of the Broad Learning System

To maintain consistency in this text, variables are represented in italics, vectors are represented in bold lowercase letters (e.g., x), and matrices are represented in bold uppercase letters (e.g., X). More generally, for any matrix $A\in R^{m\times n}$, $a^{i}\in R^{1\times n}$, $a_{j}\in R^{m\times 1}{,a}_{i,j}$ represents the element of matrix A at the ith row and jth column. The capital letter T indicates the transpose operator. Let us denote $X=\left[x^{1};x^{2};\ldots ;x^{N}\right]\in R^{N\times M}$ as the training sample matrix, where N represents the number of samples, M represents the feature dimension, and $Y=[y^{1};y^{2};\ldots ;y^{N}]\in R^{N\times C}$ as the training label matrix, where C means the number of classes.

The introduction of the Broad Learning System (BLS) provides an effective and efficient learning framework for classification and regression problems. The main advantage of BLS is its ability to map input data into a series of random feature spaces and determine output weights through optimized least squares. When new nodes or inputs arise, the training process can be extended to an incremental learning mode.

Here, a set of labeled training samples is provided to the BLS, for example, {X,Y}. Assume that BLS has n groups of feature mapping nodes; each group contains k feature mapping nodes. The feature node can be represented by $Z_{i}$.

(1) $$Z_{i}=\left[\phi_{i}\left(XW_{e_{i}}+\beta_{e_{i}}\right)\right]\in R^{N\times k},i=1,2,3,\ldots ,n$$

where $\phi_{i}(\cdot )$ represents the activation function. $\;W_{e_{i}}\in R^{M\times k}\;and\;\beta_{e_{i}}\in R^{N\times k}$ both represent randomly generated weight matrices and biases.

Connecting the n groups of feature mapping nodes together forms the feature mapping layer. Here, the feature mapping layer is represented by $Z^{n}$.

(2) $$Z^{n}=\left[Z_{1};Z_{2};Z_{3}\ldots ;Z_{n}\right]\in R^{N\times nk}$$

The feature mapping layer $Z^{n}$ is passed to enhancement nodes to construct the feature enhancement layer. The enhancement node is represented by $H_{j}$.

(3) $$H_{j}=\left[\xi_{j}\left(Z^{n}W_{h_{j}}+\beta_{h_{j}}\right)\right]\in R^{N\times b},i=1,2,3,\ldots ,n$$

where ${\xi_{j}}_{i}(\cdot )$ represents the activation function. $W_{h_{j}}\in R^{nk\times b}\;and\;\beta_{h_{j}}\in R^{N\times b}$ both represent randomly generated weight matrices and biases.

The enhancement nodes are connected together to form the enhancement layer, resulting in $H^{m}$. The enhancement layer has m groups of enhancement nodes; each group contains b nodes.

(4) $$H^{m}=\left[H_{1};H_{2};H_{3};\ldots ;H_{m}\right]\in R^{N\times mb}$$

Connecting the feature mapping layer and the enhancement layer together, the complete state matrix A can be represented as follows:

(5) $$A=\left[Z^{n}|H^{m}\right]$$

This optimization problem can be formulated as finding the regularized least squares solution of Y = AW. Hence, the BLS can be trained as follows:

(6) $$\underset{W}{\min}{\left|\left|Y-AW\right|\right|}_{2}^{2}+{\lambda \left|\left|W\right|\right|}_{2}^{2}$$

(7) $$W=A^{+}Y$$

where λ is the regularization parameter. ${W\in R}^{(nk+mb)\times C}$, $A^{+}={\left(A^{T}A+\lambda I\right)}^{-1}A^{T}$ is the Moore–Penrose pseudoinverse, and more details can be found in [5].

## 3. The Proposed Method

To enhance the robustness of the BLS framework, this section delves into a comprehensive exploration of the proposed Logarithm Kernel (LK) function. We elucidate its integration into the width learning system for training output weights, thereby yielding the reweighting framework L-BLS. At the end of this section, we will introduce a Mixture Autoencoder (MAE) that helps BLS build more representative feature nodes in a noisy label environment and two BLS versions of MAEBLS and L-MAEBLS for image data sets.

### 3.1. Logarithm Kernel (LK)

The correlation entropy between two random variables X and Y serves as a correlation measure in kernel space, as elucidated in [37,38,39,40].

(8) $$V\left(X,Y\right)=E\left[{\langle \Phi \left(X\right),\Phi \left(Y\right)\rangle }_{H}\right]=\int {\langle \Phi \left(X\right),\Phi \left(Y\right)\rangle }_{H}dF_{XY}\left(X,Y\right)$$

where $E\left[\cdot \right]$ represents the expectation operator, $F_{XY}(x,y)$ represents the joint distribution function, ${\langle \Phi (x),\Phi (y)\rangle }_{H}=\kappa (x,y)$, κ(x,y) is the Mercer kernel [21] controlled by the kernel size σ. We can clearly obtain the following equation:

(9) $$V\left(X,Y\right)=E\left(\kappa \left(X,Y\right)\right)$$

In the field of machine learning, E(κ(X,Y)) is commonly used to estimate the degree of correlation between the true values and the predicted values. In complex, noisy environments, it is important to accurately quantify the correlation between the true values and the predicted values and enhance the robustness of parameter estimation.

Non-second-order statistical measures can be elegantly defined as second-order measures in kernel space. Non-second-order statistical measures can be elegantly defined as second-order measures in kernel space. According to Property 3 provided in [39], the correlation entropy has the potential to capture the second-order and higher order statistical characteristics of the error when using a Gaussian kernel. With an appropriate kernel size setting, the second-order statistical characteristics of the error can dominate, which makes entropy-based optimization criteria a suitable choice in label noise environments as well. This paper presents a new function called the Logarithm Kernel (LK).

(10) $$\zeta \left(X,Y\right)=log\left(1+kernel\left(X,Y\right)\right)\#\left(10\right)$$

where kernel(X,Y) is equal to κ(X,Y). So unless specified, this paper employs the Gaussian kernel as the function for the Logarithm Kernel. So Equation (10) can be deduced to Equation (11).

(11) $$\zeta \left(y_{i}^{\prime },y_{i}\right)=log\left(1+{kernel}_{G}\left(y_{i}^{\prime },y_{i}\right)\right)$$

where ${kernel}_{G}(y_{i}^{\prime },y_{i})\;is\;equal\;to\;exp(-\frac{{\left||y_{i}^{\prime }-y_{i}|\right|}^{2}}{2\sigma^{2}})$ and σ>0, $y_{i}^{\prime }$ represents the predicted label, and $y_{i}$ represents the real label.

Clearly, we can obtain $V(y_{i}^{\prime },y_{i})=E({kernel}_{G}(y_{i}^{\prime },y_{i}))$. By using the Taylor series expansion to $V(y_{i}^{\prime },y_{i})$, we have

(12) $$V\left(y_{i}^{\prime },y_{i}\right)=\sum_{n=1}^{\infty }\frac{{\left(-1\right)}^{n}}{2^{n}n!}E\left[\frac{{{(y}_{i}^{\prime },y_{i})}^{2n}}{\sigma^{2n}}\right]$$

$V(y_{i}^{\prime },y_{i})$ can be regarded as the weighted sum of all the even moments of $y_{i}^{\prime }$ and $y_{i}$, with the weights of the second and higher order moments controlled by the kernel size σ. The kernel size determines the weight of the individual, even at moments when calculating the weighted sum. When σ increases, it means that we consider the difference between $y_{i}^{\prime }$ and $y_{i}$ more smoothly, and the weight of the higher order even moments decreases. This property allows the correlated entropy to better adapt to different data situations and improves its robustness in practical applications. For finite sample data, $V(y_{i}^{\prime },y_{i})$ can be approximated as follows:

(13) $$\tilde{V}\left(y_{i}^{\prime },y_{i}\right)=\frac{1}{N}\sum_{n=1}^{N}{kernel}_{G}\left(y_{i}^{\prime },y_{i}\right)$$

Similarly, each sample is introduced into ζ(X,Y)=log(1+kernel(X,Y)) and changes the following equation:

(14) $$\zeta \left(y_{i}^{\prime },y_{i}\right)=\frac{1}{N}\sum_{n=1}^{N}(1+log\left({kernel}_{G}\left(y_{i}^{\prime },y_{i}\right)\right)$$

### 3.2. The Proposed L-BLS

Based on the above motivations and to address the worse performance of ridge regression in complex label noise environments., we transform LK to a BLS-based reweighting framework, L-BLS, for training output weights.

Similar to BLS, the state matrix A can be constructed to build the feature mapping via (1)–(5). Therefore, to enhance the robustness of the BLS, incorporating the LK proposed in Section A and sample reweighting techniques into the optimization model of the BLS can be represented as follows:

(15) $$\underset{W}{\mathrm{argmax}}\left(\sum_{i=1}^{N}\log\left(1+\exp\left(-\frac{{\left|\left|y_{i}-a_{i}W\right|\right|}_{2}^{2}}{\sigma^{2}}\right)\right)-\frac{\lambda }{2}{\left|\left|W\right|\right|}_{2}^{2}\right)$$

where ${a_{i}\in R}^{L}$ represents the feature of the i-th sample among N data samples. According to our experience, if we need to find W satisfying the condition in Equation (15), we should first calculate the gradient of this equation. To simplify the calculation, let us set $\phi (W)=\sum_{i=1}^{N}log(1+exp(-\frac{{\left||y_{i}-a_{i}W|\right|}_{2}^{2}}{\sigma^{2}}))-\frac{\lambda }{2}{\left||W|\right|}_{2}^{2}$. By taking the derivative of the function ϕ(W) with respect to W, we obtain the following equation:

(16) $$\frac{\partial \phi \left(W\right)}{\partial W}=\sum_{i}^{N}\left(-\frac{\begin{array}{c} \exp\left(-\frac{{\left|\left|y_{i}-a_{i}W\right|\right|}_{2}^{2}}{\sigma^{2}}\right) \end{array}\left(a_{i}^{T}\left(a_{i}W-y_{i}\right)-\lambda W\right)}{\sigma^{2}\left(1+\exp\left(-\frac{{\left|\left|y_{i}-a_{i}W\right|\right|}_{2}^{2}}{\sigma^{2}}\right)\right)}\right)$$

To simplify subsequent calculations, $\frac{\partial \phi (W)}{\partial W}$ can be represented as follows:

(17) $$\frac{\partial \phi \left(W\right)}{\partial W}=-\frac{1}{\sigma^{2}}A^{T}U\left(AW-Y\right)-\lambda W$$

where U is equal to

(18) $$\left[\begin{array}{ccc} \frac{\begin{array}{c} \exp\left(-\frac{{\left|\left|y_{1}-a_{1}W\right|\right|}_{2}^{2}}{\sigma^{2}}\right) \end{array}}{1+\exp\left(-\frac{{\left|\left|y_{1}-a_{1}W\right|\right|}_{2}^{2}}{\sigma^{2}}\right)} & & \\ & \ddots & \\ & & \frac{\begin{array}{c} \exp\left(-\frac{{\left|\left|y_{N}-a_{N}W\right|\right|}_{2}^{2}}{\sigma^{2}}\right) \end{array}}{1+\exp\left(-\frac{{\left|\left|y_{N}-a_{N}W\right|\right|}_{2}^{2}}{\sigma^{2}}\right)} \end{array}\right]$$

Letting the partial derivative $\frac{\partial \phi (W)}{\partial W}$ be zero, W can be expressed as follows:

(19) $$\frac{\partial \phi \left(W\right)}{\partial W}=-\frac{1}{\sigma^{2}}A^{T}U\left(AW-Y\right)-\lambda W=0$$

(20) $$W={\left(A^{T}UA+\lambda \sigma^{2}E\right)}^{-1}A^{T}UY$$

Observing W and U, we can see that the right-hand side can be viewed as a function of W, so we can further rewrite them as follows:

(21) $$W=f\left(W\right)$$

(22) $${F\left(W\right)=\left(A^{T}UA+\lambda \sigma^{2}E\right)}^{-1}A^{T}UY$$

**Figure 1:**
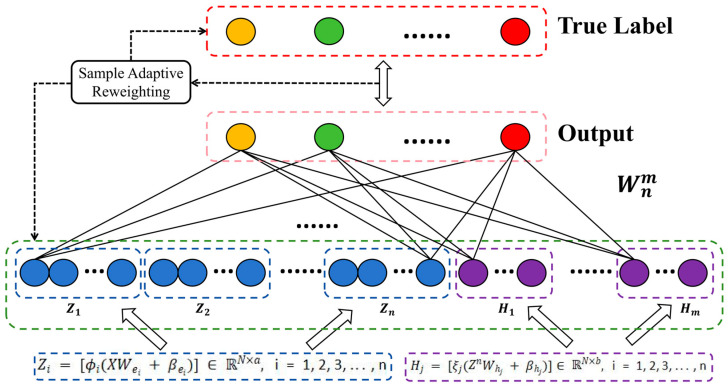
Architecture of the L-BLS.

A more intuitive L-BLS framework and detailed algorithm are shown in Figure 1 and Algorithm 1. In Algorithm 1, the weight of each sample is continuously updated by iteratively optimizing U and W. Reasonable weights are assigned to samples with the correct labels. The effectiveness of the sample weighting framework can be more intuitively presented in Section 5.
**Algorithm 1** Proposed L-BLS Sample Reweighting Framework**Input:** Training samples X with corrupted label YOutput:Output weight matrix W1: **Initialization:** parameters, regularization parameter λ, kernel size σ, termination tolerance η, and maximum iteration number T;2: for i = 1, 2, …, n do3:    $\mathrm{Random}\;W_{e_{i}}$, $\beta_{e_{i}}$;4:    $\mathrm{Calculate}\;Z_{i}=[\phi_{i}(XW_{e_{i}}+\beta_{e_{i}})]$;5: end for6: $\mathrm{Set}\;\mathrm{the}\;\mathrm{feature}\;\mathrm{mapping}\;\mathrm{group}\;Z^{n}=[Z_{1};Z_{2};Z_{3}\ldots ;Z_{n}]$;7: for j = 1, 2, …, m do8:     $\mathrm{Random}\;W_{h_{j}},\beta_{h_{j}}$;9:     $\mathrm{Calculate}\;H_{j}=[\xi_{j}(Z^{n}W_{h_{j}}+\beta_{h_{j}})]$;10: end for11: $\mathrm{Set}\;\mathrm{the}\;\mathrm{enhancement}\;\mathrm{node}\;\mathrm{group}\;H^{m}=[H_{1};H_{2};H_{3};\ldots ;H_{m}]$;12: Set the state matrix A according to Equation (5);13: for t = 1, 2, …, T do14:    Compute U according to Equation (18);15:    Update W according to Equation (20);16:    $\mathrm{if}\;{\left||W_{t}-W_{t-1}|\right|}_{2}^{2}<\eta$ then17:        break;18:    end if19: end for

### 3.3. Mixture Autoencoder

In this section, for image databases with numerous features, a novel Mixture Autoencoder (MAE) is constructed by utilizing convolutional autoencoder techniques [41] and the advantages of variational autoencoders [42]. The purpose of MAE is to help BLS and its variants create more representative feature nodes under label noise conditions [43,44].

**Figure 2:**
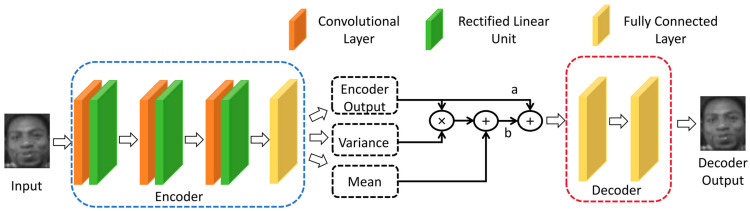
Architecture of a Mixture Autoencoder.

The encoder network consists of convolutional layers and nonlinear activation functions, which are used to extract features from the input images. The convolutional layers employ multiple convolutional kernels to perform convolutional operations on the input images, capturing the spatial structure and features of the images. Nonlinear activation functions are then applied to introduce nonlinear transformations, enhancing the expressive power and robustness of the features. The structure of the Mixture Autoencoder is shown in Figure 2. 

To achieve efficient latent representation learning, a reparameterization of EncoderOutput is performed, and reparameterization factors a and b are introduced, ensuring a + b = 1, as in Equation (23), to allocate reasonable weights. This enhances the model’s robustness while ensuring feature integrity.

(23) $$DecoderInput=aE+b\left[Variance\left(E\right)\times E+Mean\left(E\right)\right]$$

where DecoderInput represents the input to the decoder, E represents EncoderOutput, Variance(x) denotes the standard deviation of x, and Mean(x) denotes the mean of the vector x.

The decoder network is responsible for mapping the learned low-dimensional latent representation back to the original image space to verify whether the encoder part provides representative feature nodes. To simplify the model in the decoding part of MAE, a fully connected approach is adopted. Nonlinear activation functions are applied layer by layer to decode the features, reconstructing the input image through inverse transformations to recover the information of the input image to the maximum extent.

The number of convolutional layers and the size of each convolution kernel determine the receptive field and feature extraction capability of the model. More convolutional layers and larger convolution kernels can not only capture more complex and high-order features but also increase computational complexity. So, in MAE, we designed three convolutional layers with 5 × 5 convolution kernels. 

On the other hand, we encode by retaining 40%–50% of the original data features in order to provide the most representative features of the image to BLS during MAE training. The entire decoder design gradually maps low-dimensional features back to high-dimensional features through linear layers and ReLU activation functions. The original input data can be efficiently reconstructed by gradually increasing the dimensionality and complexity of the data. In the process of reconstructing the original image, if the decoder’s reconstruction step size is too small, it will increase the uncertainty and complexity of the decoding process. In this paper, we gradually reconstruct the original data through two stages of similar step size. This can improve the stability of the decoder while reducing the uncertainty and complexity of the decoder. In addition, the encoding and decoding methods of the convolutional layer and the fully connected layer determine the choice of subsequent parameters to a certain extent. 

Here are the detailed parameter settings, as shown in Table 1 and Table 2. We use ${Conv}_{i}$ and ${FullyConnected}_{i}$ to represent the i-th convolutional layer and the i-th fully connected layer.

This process enhances the feature extraction capability of BLS, strengthens BLS’s understanding and utilization of data, and provides a more reliable foundation and support for subsequent applications. In any version of BLS, MAE can be used to achieve high-quality extraction of image features.

### 3.4. The Proposed MAEBLS and L-MAEBLS

**Figure 3:**
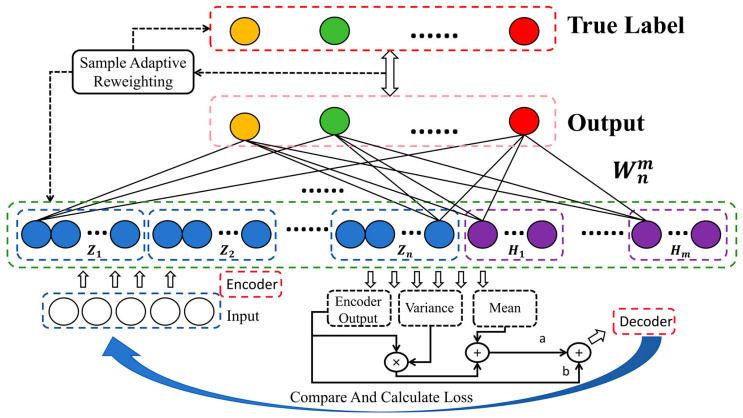
Architecture of the L-MAEBLS.

On the image database, based on BLS, LK, and MAE, we developed MAEBLS and L-MAEBLS. As can be seen from Figure 3, we embed MAE into the feature layer of BLS and use MAE to encode complex image data to help BLS build more representative feature nodes to obtain MAEBLS. At the same time, the decoder is used to verify the effectiveness of the encoder. Based on MAEBLS, transform LK to a MAEBLS-based reweighting framework for training output weights to obtain L-MAEBLS. This enables BLS to focus more on valuable features, thereby alleviating the performance degradation caused by insufficient feature extraction capabilities when label noise exists. In Section 5, the experiment results will validate our statement.

(24) $$EncoderOutput=MAE\left(data\right)$$

## 4. Proof of Robustness

The method proposed in this paper demonstrates impressive robustness; thus, its inherent mechanism for robustness to label noise will be proved in this section. On one hand, we can approximate $\zeta (y_{i}^{\prime },y_{i})$ as ζ(e), where $e={\left||y_{i}^{\prime }-y_{i}|\right|}^{2}$. We can observe that ζ(e) is bounded, smooth, and non-convex loss. Therefore, ζ(e) exhibits robustness under noisy conditions. On the other hand, we will proceed to demonstrate the robustness mechanism of the method proposed in this paper.

**Theorem 1.**  *Through L-BLS, normal samples are assigned larger weights, while noisy samples are assigned smaller weights. Therefore, L-BLS can be more robust than BLS.*

**Proof.**  The error term of the robust width learning model L-BLS proposed in this paper can be regarded as $E=\{log(1+exp(-e_{1}^{2})),log(1+exp(-e_{2}^{2})),\ldots \ldots ,log(1+exp(-e_{N}^{2}))$. Let the boundary for determining whether a sample is corrupted be θ. If $log(1+exp(-e_{i}^{2}))$ < θ, the i-th sample is considered an intact sample. Otherwise, it is considered a corrupted (containing label noise) sample. We can set the weights of the k-th sample in BLS, and L-BLS are assigned as follows:

(25) $$W_{BLS}^{k}=\frac{e_{k}^{2}}{\sum_{i=1}^{N}e_{i}^{2}}$$

(26) $$W_{L-BLS}^{k}=\frac{L_{k}^{\;}}{\sum_{i=1}^{N}L_{i}^{\;}}$$

where, for ease of representation and understanding, let us set $L_{k}^{\;}=log(1+exp(-e_{k}^{2}))$. We define $\delta^{k}=\frac{W_{L-BLS}^{k}}{W_{BLS}^{k}}$. Substituting the above equation into Equation (30), we get

(27) $$\delta^{k}=\frac{W_{BLS}^{k}}{W_{L-BLS}^{k}}=\frac{L_{k}^{\;}\sum_{i=1}^{N}e_{i}^{2}}{e_{k}^{2}\sum_{i=1}^{N}L_{i}^{\;}}$$

Furthermore, by normalizing the Cauchy–Schwarz inequality [30] to the above equation, we obtain the following equation:

(28) $$\sum_{i=1}^{N}({\frac{1}{N})}^{\frac{1}{2}}\frac{L_{k}^{\;}e_{i}^{2}}{L_{i}^{\;}e_{k}^{2}}\leq \delta^{k}$$

(29) $$\sum_{i=1}^{N}({\frac{1}{N}\frac{L_{k}^{2}e_{i}^{4}}{L_{i}^{2}e_{k}^{4}})}^{\frac{1}{2}}\leq \delta^{k}$$

An obvious fact is that the error of noisy data is much larger than that of intact data. Therefore, when $e_{k}$ belongs to normal training samples, we obtain the following equation:

(30) $$\sum_{i=1}^{N}\frac{L_{k}^{2}e_{i}^{4}}{L_{i}^{2}e_{k}^{4}}>N$$

Further derivation leads to the following equation:

(31) $$\delta^{k}=\frac{{W_{L-BLS}^{k}}_{\;}^{\;}}{W_{BLS}^{k}}>1$$

This implies that in L-BLS, compared to the base width learning system, normal data can be assigned larger weights. Thus, all training data can be concluded as $\sum_{i=1}^{N}W_{BLS}^{i}=\sum_{i=1}^{N}W_{L-BLS}^{i}=1$. Thus, we can derive the following equation:

(32) $$\sum_{{|e}_{i}^{\;}|<\theta }W_{L-BLS}^{i}>\sum_{{{|e}_{i}^{\;}|<\theta }_{\;}}W_{BLS}^{i}$$

(33) $$\sum_{{|e}_{i}^{\;}|>\theta }W_{L-BLS}^{i}<\sum_{{{|e}_{i}^{\;}|>\theta }_{\;}}W_{BLS}^{i}$$

Thus, this proof is finished. □

## 5. Experimental Results

In this section, the performance of the proposed L-BLS, L-MAEBLS, and MAEBLS for classifying data with label noise was evaluated through extensive experiments. Accuracy (ACC) was chosen as the evaluation metric.

(34) $$ACC=\frac{1}{N}\sum_{n=1}^{N}\psi \left(y_{i}^{\prime },y_{i}\right)$$

where ψ(a,b) is a function that computes the number of correctly classified samples. Unless otherwise stated, all experiments were conducted using Python 3.10 on a computer equipped with an Intel i7 2.5-GHz CPU and 16-GB RAM.

### 5.1. Dataset Selection and Parameter Settings

Our experiments utilized six datasets from the UC Irvine (UCI) Machine Learning Repository [45] and three image classification datasets: Coil20 [46], ORL [47], and UMIST. Their features and partitions are shown in Table 3.

In the UCI datasets, we selected BLS [5] and four robust BLS models, including WBLS [23], C-BLS [26], ENBLS [22], and GRBLS [27], as comparison methods. On the image datasets, we constructed MAEBLS and L-MAEBLS based on the above methods and compared them with their original versions to demonstrate the ability of MAE in feature extraction under label noise [48,49,50]. To ensure fairness, we conducted grid searches within the same range to search for common parameters of the comparison methods in order to obtain the best performance.

Commonly used parameters include the number of feature mapping groups $N_{\omega }$, the number of feature mapping nodes per group $N_{f}$, the number of enhancement nodes $N_{e}$, and the L2 regularization parameter λ.

More specifically, for each UCI dataset, the search range for $N_{f}$ and $N_{\omega }$ is [1,15], with a step size of 2. The search range for $N_{e}$ is [10,50], with a step size of 5. Search for the L2 regularization parameter within the range ${[2^{-30},2^{-25},\ldots ,2}^{0}]$. For each image dataset, the search range for $N_{f}$ and $N_{\omega }$ is [10, 50], with a step size of 5. Besides, the search range for $N_{e}$ is [1000, 5000], with a step size of 1000. The search range for the L2 regularization parameter λ is set to be the same as the corresponding range for the UCI datasets.

In the comparison methods, refer to the corresponding methodological papers, in the Huber–WBLS model, the positive adjustable parameter is set to 1.345. For the C-BLS model, the kernel size; for the ENBLS model, the L1 regularization parameter $\lambda_{1}$ and the L2 regularization parameter $\lambda_{2}$; for the GRBLS model, the regularization factor for the manifold term; and for the L-BLS model, the kernel width are all searched within the range of ${[2^{-30},2^{-25},\ldots 2}^{0}]$.

Additionally, to eliminate the scale effect, we normalize the attributes of the datasets to [–1, 1]. For the UCI datasets, their attributes are normalized individually. For the three 8-bit grayscale image datasets, all attributes are divided by 127.5 and then subtracted by 1. Each dataset undergoes 50 repeated processes with all comparison methods, using the corresponding fixed optimal parameters to ensure the stability and reliability of the results.

### 5.2. Noise Modeling

People generally believe that labels always have a greater impact on the modeling process. In the model learning process, since the importance of each feature to model learning varies, label noise tends to have a more profound and detrimental effect than feature noise.

Ghosh et al. [51] proposed that label noise can be represented as follows:

(35) $$\overset{\sim }{y}=\left\{\begin{array}{c} \;y\;,\;\;\;\;\;\;\;\;\;\;\;\;\;\;\;\mathrm{with}\;\mathrm{probability}\;\;1-\eta \\ y_{j},j\epsilon \left[N\right],y_{j}\neq y,\mathrm{with}\;\mathrm{probability}\;\;\eta_{j}=\frac{\eta }{N-1} \end{array}\right.$$

where η represents the noise ratio, and satisfies $\eta =\sum \eta_{j}$. N represents the total number of classes. When η is a constant, $\eta_{j}$ can be represented as $\eta_{j}=\frac{\eta }{N-1}$. In this case, the type of label noise is symmetric or uniform noise. Otherwise, the noise type is asymmetric noise, where the true labels are randomly flipped to another class.

To be more realistic, in our experiments, we chose uniform label noise to simulate common noise situations. As for the process of mislabeling, it is completely random, with equal probabilities for all other classes.

### 5.3. Performance Evaluation on Data with Label Noise

In this section, the comparison results of the above methods on different UCI datasets under different pollution rates (η=0%, η=10%, …, η=50%) are compared. The results are shown in Table 4 and Table 5 as the average values ± STD (%), with the best results highlighted in bold. 

**Figure 4:**
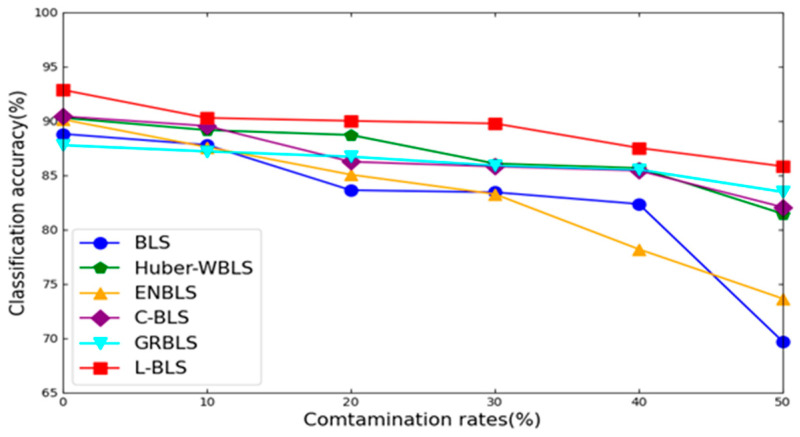
Classification performance trends of different algorithms on the Iris dataset corrupted by noise of diverse levels.

**Figure 5:**
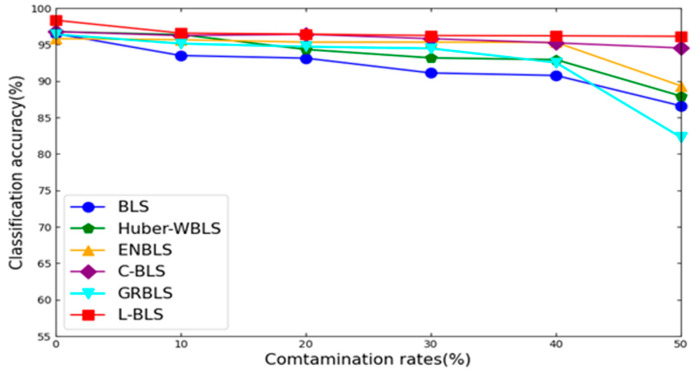
Classification performance trends of different algorithms on the Ecoil dataset corrupted by noise of diverse levels.

After thorough experimentation, we have confirmed the superior performance of L-BLS. Analyzing Table 4 and Table 5, we can make the following observations: First of all, across most UCI datasets, L-BLS outperforms competitors at various contamination levels, especially at higher noise levels like 40% and 50%. For instance, on the Wine dataset with 40% label noise, L-BLS achieves an impressive average accuracy of 94.25% with a minimal standard deviation (STD) of 0.96, while other methods struggle to reach 90% accuracy. This underscores L-BLS’s robustness to label noise. Figure 4 and Figure 5 provide a visual comparison of method trends on the Iris and Ecoil datasets. L-BLS outperforms other methods under any contamination rate conditions. In the Iris dataset, the accuracy of the L-BLS method is always above 96%, which is difficult to achieve with other methods. In particular, in the Ecoil dataset, when other methods are seriously affected by label noise, the L-BLS method can still remain stable and be far ahead of other methods. We can conclude that as the contamination rate increases, L-BLS maintains an acceptable accuracy drop, while the accuracy of other methods drops significantly. Second, L-BLS surpasses all comparison methods on COIL20, ORL, and UMIST datasets, except for L-MAEBLS, even maintaining acceptable performance degradation under severe contamination rates. This shows that L-BLS can still demonstrate strong robustness on image datasets. Third, on most clean datasets, L-BLS demonstrates superior performance compared to standard BLS and other methods, suggesting its capability to reweight and detect samples, further enhancing its performance. Overall, L-BLS consistently outperforms other methods across different scenarios, with acceptable performance even under high contamination rates. These comprehensive results indicate promising applications for L-BLS. 

Notably, L-BLS consistently exhibits superior performance and accuracy across different contamination rates, especially under high noise levels, showcasing its robustness in label noise environments for effective classification tasks.

### 5.4. The Performance of L-BLS Combined with MAE

**Figure 6:**
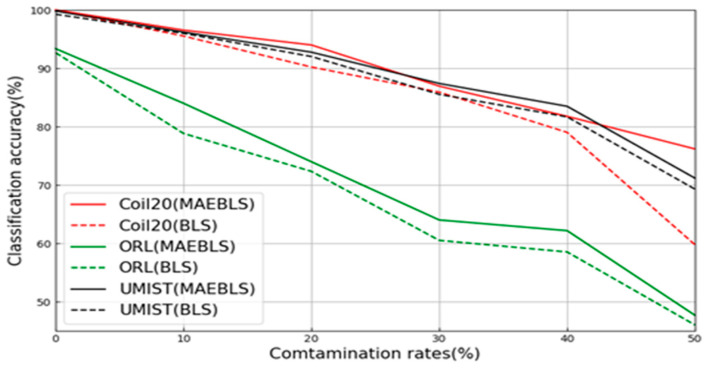
Classification performance of MAEBLS corrupted by noise of diverse levels in three image datasets.

**Figure 7:**
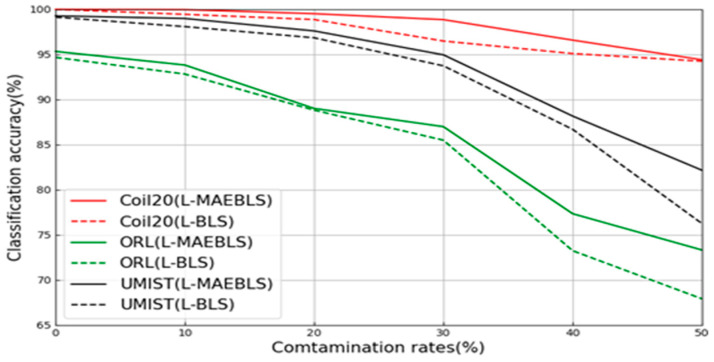
Classification performance of MAEBLS corrupted by noise of diverse levels in three image datasets.

**Figure 8:**
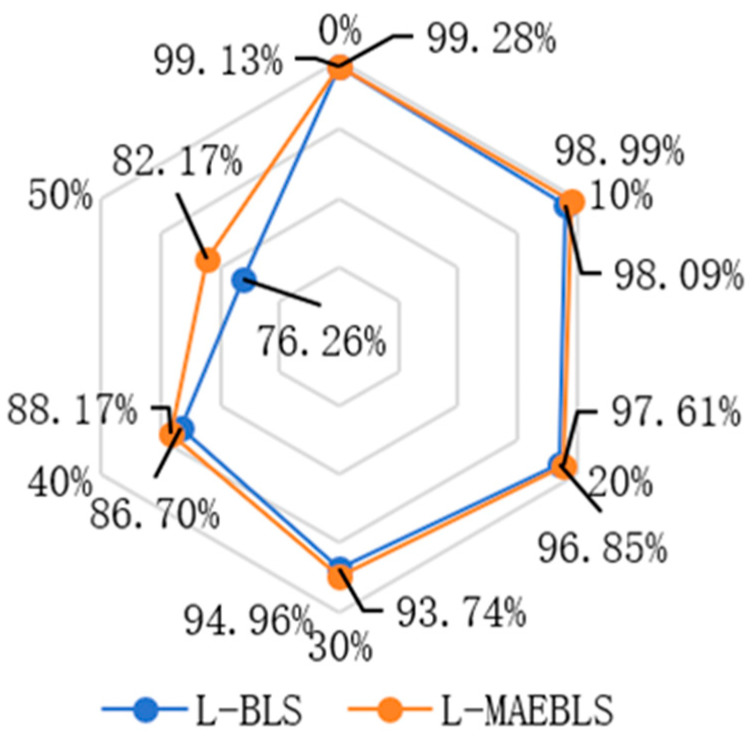
Performance of L-MAEBLS on the UMIST database.

**Figure 9:**
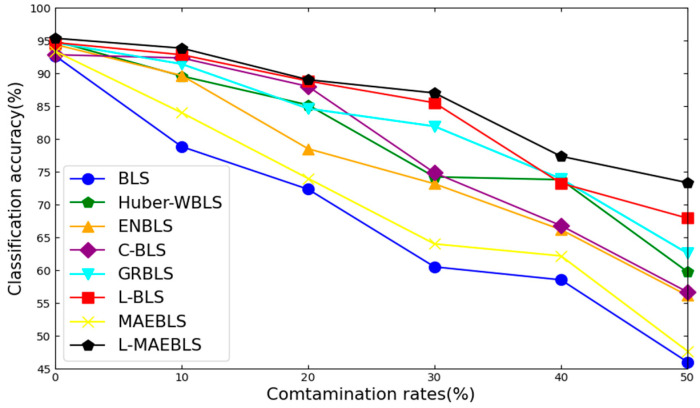
Classification performance trends of different algorithms on the ORL database corrupted by noise of diverse levels.

In the image dataset, in order to verify the MAE’s ability to enhance feature extraction and robustness under label noise conditions in the wide learning system, we added MAEBLS and L-MAEBLS to the experimental part and compared them with their corresponding BLS versions. In the COIL20, ORL, and UMIST datasets, MAEBLS shows more powerful feature extraction capabilities than BLS, and L-MAEBLS shows more powerful feature extraction capabilities than L-BLS. Notably, L-MAEBLS exhibited even greater robustness. Key findings from Table 5 are as follows: 

First of all, for the COIL20, ORL, and UMIST datasets, the proposed MAEBLS and L-MAEBLS are superior to BLS and L-BLS at most pollution rates. From Figure 6 and Figure 7, it can be seen intuitively that MAE can effectively help BLS construct feature nodes. In particular, as shown in Figure 8, L-MAEBLS shows a significant improvement over BLS in accuracy. When η=50%, L-MAEBLS can improve the accuracy by 5.91% compared with L-BLS, and other noise conditions are also significantly improved. Second, in the above three image datasets, L-MAEBLS outperforms all other comparison methods in most cases. From Figure 9, we can intuitively see the excellent performance of L-MAEBLS on the ORL database, especially when there is 50% label noise. The average accuracy of L-MAEBLS is 73.33%, with a standard deviation of only 0.65, while other methods struggle to achieve an accuracy of 70%.

### 5.5. The Effectiveness of L-BLS

In order to further verify the effectiveness of the reweighting framework L-BLS, in this section, we take the COIL20 dataset under the condition of symmetric label noise with a contamination rate of η=30% as an example. We plot the sum of squares of residuals for each sample to visually evaluate the effectiveness of the proposed method. Since one-hot encoding is used to encode the labels of the samples, it is necessary to calculate the residuals for each element of each sample and then sum the squares of the residuals for each element.

(36) $$SUM\left({\left(Y^{\prime }-Y\right)}^{2}\right)$$

where SUM(A) represents the sum of rows in matrix A.

**Figure 10:**
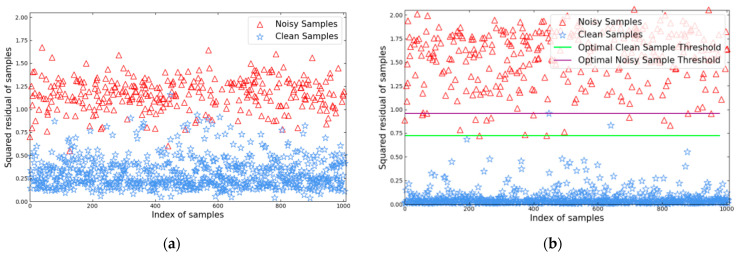
Residual of each training sample in the COIL20 database at contamination rate η = 30%. (**a**) First iteration. (**b**) Optimal iteration.

The residuals and their squares for each sample in the initial iteration and at the optimal iteration are shown in Figure 10a.

From the results depicted in Figure 10a, it can be inferred that there exists an overlapping region, making it challenging to differentiate between clean and noisy samples. Hence, employing an iterative sample identification approach is more reasonable. Initially, only a few samples are distinctly identified as clean or noisy, with appropriate element weights, while the remaining samples are considered unidentified samples with moderate weights, effectively avoiding the negative impact of misjudgment. As the iterations progress, the squared errors of clean samples gradually decrease, while those of noisy samples gradually increase, facilitating more accurate sample identification.

Observing the results from Figure 10b, it can be noted that in the optimal iteration process, almost all clean samples are correctly identified, while the adverse effects of some noisy samples are effectively suppressed.

### 5.6. Statistical Analysis

In this section, we provide an analysis using the Friedman test to evaluate the statistical significance of the differences between the specified methods on the UCI datasets and image datasets.

First, we use the Friedman test with a confidence level of α = 0.1 to test the overall performance of different algorithms. As shown in Table 6, we can see that on the UCI dataset, our proposed L-BLS ranks the highest. C-BLS is second, while Huber-WBLS, ENBLS, and GRBLS perform better than the standard BLS. In the image dataset, as shown in Table 7, our proposed L-MAEBLS and L-BLS rank first and second, respectively, and our proposed MAEBLS also shows an improvement in ranking compared to BLS.

Second, as noise level is a critical factor affecting classifier performance, we employed the Friedman test at a confidence level of α=0.1 to test the statistical differences of the complete set of algorithms under various noise levels. Table 8 and Table 9 display the results of the Friedman tests for the UCI datasets and the image datasets, respectively. In Table 8, the participants included BLS, ENBLS, C-BLS, GRBLS, Huber-WBLS, and L-BLS. Building on these methods, MAEBLS and L-MAEBLS also took part in the Friedman test, as shown in Table 9.

This paper conducts comparative experiments on the current advanced, robust BLS family methods under a more rigorous grid search scope. In the vast majority of cases, the *p*-values are less than 0.1, which implies that the statistical differences among the mentioned methods on the UCI database and image databases are significant under various noise levels.

## 6. Conclusions

In order to enhance the robustness and feature extraction ability of BLS in label noisy environments, we proposed in this paper, three versions of BLS: L-BLS, L-MAEBLS, and MAEBLS for modeling label noisy data. Based on the Gaussian kernel LK function, a new sample reweighting framework, L-BLS, is derived. Then the loss function and regularization term of the L-BLS classification problem are comprehensively studied. And for the image dataset, a mixture autoencoder MAE is constructed based on the convolutional autoencoder technology and the variational autoencoder technology, and the corresponding L-MAEBLS and MAEBLS versions are developed. Then the robustness and effectiveness of L-BLS are proven. The proposed L-BLS and L-MAEBLS can accurately simulate the loss distribution and are insensitive to label noise. MAE can provide more representative feature nodes for the corresponding BLS version. The experimental results show that the proposed L-BLS does achieve better performance in terms of robustness and effectiveness, and MAE does improve the feature extraction ability of the corresponding BLS version under label noise conditions. 

This paper mainly focuses on the classification problem of BLS without considering the robust regression problem, which will be our future work.

## Figures and Tables

**Table 1:** Convolutional layer parameter settings.

Name of Layer	Input Channel	Output Channel	Kernel Size
${Conv}_{1}$	1	2	5 × 5
${Conv}_{2}$	2	4	5 × 5
${Conv}_{3}$	4	1	5 × 5

**Table 2:** Fully connected layer parameter settings.

Name of Layer	Input Features	Output Features
${FullyConnected}_{1}$	20	20
${FullyConnected}_{2}$	400	700
${FullyConnected}_{3}$	700	1024

**Table 3:** Characteristics of the selected datasets.

Datasets	Attributes	Classes	No. of Instances	
			Training	Testing
Statlog (Heart)	13	2	162	108
Iris	4	3	90	60
Wine	13	3	107	71
Balance scale	4	3	375	250
Zoo	16	7	61	40
Ecoil	8	8	202	134
Coil20	1024	20	1008	432
ORL	1024	40	280	120
UMIST	1024	20	345	230

**Table 4:** The classification results of different methods on different test sets of UCI databases.

Datasets	Algorithms	η = 0%	η = 10%	η = 20%	η = 30%	η = 40%	η = 50%
		Aver ± STD (%)					
Statlog (Heart)	BLS	80.74 ± 2.06	77.78 ± 1.14	74.72 ± 3.27	71.43 ± 2.24	66.64 ± 3.16	60.92 ± 3.07
	Huber-WBLS	82.23 ± 0.89	82.04 ± 0.88	79.69 ± 0.84	77.90 ± 0.94	67.53 ± 0.97	63.27 ± 0.66
	ENBLS	83.14 ± 1.08	82.22 ± 0.69	80.19 ± 0.45	73.19 ± 2.44	67.24 ± 1.50	54.44 ± 0.37
	C-BLS	82.96 ± 0.94	81.39 ± 0.49	81.02 ± 0.62	80.09 ± 0.75	76.85 ± 1.01	66.29 ± 1.31
	GRBLS	81.12 ± 6.13	77.99 + 9.12	72.37 ± 9.26	71.72 ± 9.20	68.24 ± 8.24	67.81 ± 8.99
	L-BLS	85.93 ± 1.36	83.24 ± 0.48	82.96 ± 0.94	81.67 ± 0.37	77.59 ± 1.36	73.33 ± 0.37
Iris	BLS	96.50 ± 0.50	93.49 ± 1.71	93.13 ± 1.32	91.10 ± 1.31	90.76 ± 1.77	86.59 ± 3.07
	Huber-WBLS	96.77 ± 1.47	96.36 ± 0.67	94.33 ± 1.11	93.17 ± 0.89	92.93 ± 1.92	87.93 ± 5.09
	ENBLS	95.80 ± 0.96	95.66 ± 0.82	95.33 ± 0.11	95.33 ± 1.45	95.30 ± 0.82	89.33 ± 2.81
	C-BLS	96.77 ± 1.47	96.23 ± 0.73	96.40 ± 0.61	95.80 ± 1.06	95.23 ± 1.25	94.53 ± 2.11
	GRBLS	96.39 ± 6.67	95.13 ± 7.74	94.70 ± 6.23	94.47 ± 8.22	92.53 ± 5.85	82.27 ± 8.36
	L-BLS	98.33 ± 1.11	96.56 ± 0.39	96.43 ± 0.57	96.23 ± 0.98	96.20 ± 0.74	96.13 ± 2.65
Wine	BLS	95.27 ± 1.61	94.08 ± 0.74	93.09 ± 1.16	86.14 ± 0.71	77.15 ± 7.29	60.98 ± 4.28
	Huber-WBLS	98.09 ± 0.67	97.25 ± 0.84	96.90 ± 0.85	93.66 ± 0.94	88.17 ± 1.31	77.82 ± 1.72
	ENBLS	96.19 ± 1.27	95.77 ± 0.85	94.36 ± 1.28	91.41 ± 1.85	80.98 ± 1.84	69.98 ± 7.92
	C-BLS	97.54 ± 0.61	96.95 ± 1.13	95.85 ± 0.99	92.92 ± 1.03	89.84 ± 1.04	74.87 ± 1.76
	GRBLS	97.01 ± 1.18	96.25 ± 9.37	95.18 ± 9.68	94.85 ± 3.70	93.94 ± 3.14	75.52 ± 10.16
	L-BLS	98.73 ± 0.42	98.62 ± 1.19	98.62 ± 0.53	95.74 ± 2.09	94.25 ± 1.96	94.39 ± 9.69
Balance scale	BLS	92.44 ± 0.22	90.99 ± 0.60	90.60 ± 1.39	88.35 ± 0.87	87.29 ± 1.39	78.51 ± 1.72
	Huber-WBLS	94.40 ± 0.31	94.38 ± 0.09	93.82 ± 0.53	91.22 ± 0.73	90.86 ± 1.97	86.76 ± 0.63
	ENBLS	93.08 ± 1.71	92.02 ± 1.35	91.12 ± 2.40	90.64 ± 2.65	89.76 ± 2.85	85.07 ± 2.45
	C-BLS	93.72 ± 0.18	91.60 ± 0.36	91.44 ± 0.51	90.60 ± 0.45	88.12 ± 0.18	87.04 ± 1.22
	GRBLS	91.44 ± 1.22	91.34 ± 3.13	91.12 ± 1.85	89.65 ± 1.50	88.96 ± 1.93	85.45 ± 2.36
	L-BLS	94.88 ± 0.64	94.68 ± 0.18	94.56 ± 0.41	94.19 ± 0.27	93.08 ± 0.81	89.40 ± 0.76
Zoo	BLS	96.10 ± 2.01	90.64 ± 2.73	87.45 ± 6.35	84.15 ± 2.63	81.50 ± 1.22	66.90 ± 4.08
	Huber-WBLS	97.56 ± 4.22	92.20 ± 1.46	89.51 ± 2.68	88.54 ± 2.20	85.61 ± 3.17	73.90 ± 2.45
	ENBLS	93.90 ± 3.75	92.15 ± 4.01	90.41 ± 4.04	91.50 ± 4.15	83.05 ± 2.36	77.05 ± 5.58
	C-BLS	98.71 ± 1.25	97.30 ± 2.17	95.60 ± 1.78	92.39 ± 2.49	87.40 ± 0.70	75.05 ± 5.39
	GRBLS	97.05 ± 1.63	96.80 ± 1.42	96.15 ± 1.67	93.85 ± 3.09	85.50 ± 2.92	83.65 ± 2.13
	L-BLS	98.95 ± 1.23	97.45 ± 0.35	97.25 ± 0.75	96.25 ± 2.14	90.75 ± 3.01	85.10 ± 1.93
Ecoil	BLS	88.82 ± 1.11	87.81 ± 0.54	83.64 ± 1.59	83.45 ± 1.15	82.37 ± 1.76	69.70 ± 3.08
	Huber-WBLS	90.30 ± 1.09	89.17 ± 0.69	88.72 ± 1.32	86.09 ± 1.64	85.68 ± 1.69	81.47 ± 5.11
	ENBLS	90.15 ± 1.15	87.61 ± 1.34	85.07 ± 2.45	83.28 ± 1.17	78.21 ± 3.64	73.66 ± 2.94
	C-BLS	90.44 ± 0.56	89.55 ± 0.75	86.26 ± 0.89	85.82 ± 1.49	85.44 ± 1.07	82.08 ± 1.29
	GRBLS	87.78 ± 0.68	87.21 ± 1.14	86.72 ± 0.68	85.91 ± 1.57	85.53 ± 1.07	83.48 ± 2.27
	L-BLS	92.87 ± 1.79	90.29 ± 1.24	90.02 ± 1.01	89.78 ± 1.05	87.53 ± 1.06	85.84 ± 1.11

**Table 5:** The classification results of different methods on different test sets of Images databases.

Datasets	Algorithms	η = 0%	η = 10%	η = 20%	η = 30%	η = 40%	η = 50%
		Aver ± STD (%)					
COIL20	BLS	99.95 ± 0.03	95.52 ± 0.76	90.21 ± 1.04	85.91 ± 0.73	79.01 ± 1.28	59.75 ± 1.34
	Huber-WBLS	100 ± 0.00	99.54 ± 0.32	96.81 ± 0.63	96.03 ± 0.57	94.89 ± 0.62	92.52 ± 0.84
	ENBLS	99.19 ± 0.46	98.89 ± 0.32	95.54 ± 0.58	94.62 ± 1.04	90.06 ± 0.97	89.98 ± 1.28
	C-BLS	100 ± 0.00	95.61 ± 0.23	95.33 ± 0.09	92.10 ± 0.23	91.82 ± 0.31	90.02 ± 0.31
	GRBLS	97.85 ± 0.21	97.21 ± 0.34	97.16 ± 0.21	94.73 ± 0.76	94.08 ± 0.58	88.22 ± 0.62
	L-BLS	100 ± 0.00	99.45 ± 0.15	98.87 ± 0.27	96.49 ± 0.67	95.10 ± 0.32	94.25 ± 0.97
	MAEBLS	100 ± 0.00	96.54 ± 0.46	93.99 ± 0.39	86.93 ± 0.50	81.76 ± 0.39	76.17 ± 0.66
	L-MAEBLS	100 ± 0.00	99.97 ± 0.19	99.52 ± 0.15	98.86 ± 0.23	96.59 ± 0.32	94.41 ± 0.31
ORL	BLS	92.67 ± 1.78	78.83 ± 0.66	72.33 ± 1.78	60.50 ± 2.33	58.52 ± 2.20	46.00 ± 1.61
	Huber-WBLS	94.92 ± 1.15	89.58 ± 0.81	85.17 ± 0.95	74.21 ± 0.98	73.79 ± 2.12	59.75 ± 1.57
	ENBLS	94.39 ± 1.78	89.67 ± 2.72	78.50 ± 2.20	73.16 ± 2.07	66.17 ± 1.72	56.14 ± 1.13
	C-BLS	92.83 ± 0.41	92.34 ± 0.33	88.00 ± 0.67	74.83 ± 1.10	66.79 ± 1.05	56.67 ± 1.17
	GRBLS	94.67 ± 0.67	91.42 ± 0.84	84.63 ± 0.71	81.92 ± 1.10	73.92 ± 1.18	62.58 ± 1.37
	L-BLS	94.68 ± 0.66	92.83 ± 1.13	88.83 ± 0.91	85.50 ± 1.67	73.25 ± 1.22	67.92 ± 2.30
	MAEBLS	93.36 ± 1.05	84.00 ± 1.43	73.98 ± 1.22	64.00 ± 1.86	62.17 ± 2.61	47.66 ± 1.33
	L-MAEBLS	95.34 ± 0.85	93.83 ± 0.67	89.02 ± 0.82	87.00 ± 1.30	77.35 ± 0.97	73.33 ± 0.65
UMIST	BLS	99.26 ± 0.20	95.98 ± 0.75	92.01 ± 1.25	85.53 ± 1.30	81.67 ± 1.51	69.27 ± 1.65
	Huber-WBLS	97.81 ± 0.36	97.22 ± 0.43	94.89 ± 0.93	89.95 ± 0.94	82.68 ± 1.08	74.30 ± 1.25
	ENBLS	96.40 ± 0.29	96.37 ± 0.41	93.90 ± 0.40	92.01 ± 0.38	96.13 ± 0.92	75.65 ± 4.76
	C-BLS	99.12 ± 0.85	97.89 ± 0.29	96.52 ± 0.67	93.04 ± 0.92	82.61 ± 0.82	76.09 ± 0.91
	GRBLS	96.61 ± 0.64	95.32 ± 0.57	93.74 ± 0.63	90.13 ± 0.62	86.09 ± 0.67	75.69 ± 0.99
	L-BLS	99.13 ± 0.11	98.09 ± 0.28	96.85 ± 0.50	93.74 ± 0.89	86.70 ± 0.59	76.26 ± 1.09
	MAEBLS	99.80 ± 0.18	96.23 ± 0.41	92.75 ± 1.08	87.39 ± 0.61	83.48 ± 1.85	71.16 ± 1.82
	L-MAEBLS	99.28 ± 0.21	98.99 ± 0.24	97.61 ± 0.22	94.96 ± 0.29	88.17 ± 1.39	82.17 ± 0.72

**Table 6:** Average rankings of different algorithms in classification accuracy in UCI databases.

	BLS	Huber-WBLS	ENBLS	C-BLS	GRBLS	L-BLS
Accuracy ranks	5.6667	3.1528	4.2639	2.9583	3.9583	1
Chi-sq	125.19					
*p*-value	$2.49293\;\times \;10^{-25}$					

**Table 7:** Average rankings of different algorithms in classification accuracy in image databases.

	BLS	Huber-WBLS	ENBLS	C-BLS	GRBLS	L-BLS	MAEBLS	L-MAEBLS
Accuracy Ranks	7.5556	4.1111	5.2222	4.5556	4.7222	2.4444	6.1667	1.2222
Chi-sq	84.54							
*p*-value	$1.62493\;\times \;10^{-15}$							

**Table 8:** Statistical testing of classification accuracy on the UCI databases.

Noise Level	Chi-sq	*p*-Value
r = 0%	23.56	${2.63\times 10}^{-4}$
r = 10%	23.14	${3.17\times 10}^{-3}$
r = 20%	21.84	${5.61\times 10}^{-4}$
r = 30%	21.08	${7.93\times 10}^{-4}$
r = 40%	21.62	${6.18\times 10}^{-4}$
r = 50%	21.14	${7.61\times 10}^{-4}$

**Table 9:** Statistical testing of classification accuracy on the image database.

Noise Level	Chi-sq	*p*-Value
r = 0%	10.50	0.1619
r = 10%	16.00	0.0251
r = 20%	19.11	0.0784
r = 30%	18.78	0.0089
r = 40%	15.44	0.0307
r = 50%	19.01	0.0081

## Data Availability

Data are contained within the article.
